# Diverse Aggregation Kinetics Predicted by a Coarse-Grained Peptide Model

**DOI:** 10.1021/acs.jpcb.1c00290

**Published:** 2021-07-12

**Authors:** Beata Szała-Mendyk, Andrzej Molski

**Affiliations:** Faculty of Chemistry, Adam Mickiewicz University in Poznań, Umultowska 89b, 61-614 Poznań, Poland

## Abstract

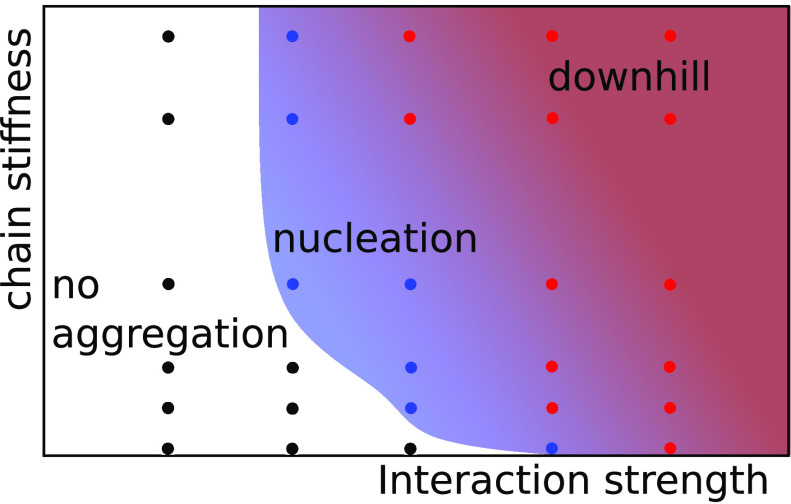

Protein and peptide aggregation is a ubiquitous phenomenon with implications in medicine, pharmaceutical industry, and materials science. An important issue in peptide aggregation is the molecular mechanism of aggregate nucleation and growth. In many experimental studies, sigmoidal kinetics curves show a clear lag phase ascribed to nucleation; however, experimental studies also show downhill kinetics curves, where the monomers decay continuously and no lag phase can be seen. In this work, we study peptide aggregation kinetics using a coarse-grained implicit solvent model introduced in our previous work. Our simulations explore the hypothesis that the interplay between interchain attraction and intrachain bending stiffness controls the aggregation kinetics and transient aggregate morphologies. Indeed, our model reproduces the aggregation modes seen in experiment: no observed aggregation, nucleated aggregation, and rapid downhill aggregation. We find that the interaction strength is the primary parameter determining the aggregation mode, whereas the stiffness is a secondary parameter modulating the transient morphologies and aggregation rates: more attractive and stiff chains aggregate more rapidly and the transient morphologies are more ordered. We also explore the effects of the initial monomer concentration and the chain length. As the concentration decreases, the aggregation mode shifts from downhill to nucleated and no-aggregation. This concentration effect is in line with an experimental observation that the transition between downhill and nucleated kinetics is concentration-dependent. We find that longer peptides can aggregate at conditions where short peptides do not aggregate at all. It supports an experimental observation that the elongation of a homopeptide, e.g., polyglutamine, can increase the aggregation propensity.

## 1 Introduction

Protein and peptide aggregation is a ubiquitous phenomenon with implications in medicine,^1^ pharamaceutical industry,^2^ and materials science.^3^ Despite intense experimental and theoretical work, peptide aggregation is not yet completely understood.^4,5^ An important issue in peptide aggregation is the molecular mechanism of aggregate nucleation and growth. In many experimental studies, sigmoidal kinetics curves show a clear lag phase ascribed to nucleation, where the initially formed small oligomers equilibrate with free monomers until aggregates of the critical size are formed.^5^ However, experimental studies also show downhill kinetics curves, where the monomers decay continuously and no lag phase can be seen. Examples of downhill aggregation include the SH3 domain of α-spectrin (Spc-SH3),^6^ transthyretin (TTR),^7,8^ human serum albumin (HSA),^9^ bovine serum albumin (BSA), and α-chymotrypsinogen A (α-chymo).^10^

The transition from the nucleated to downhill kinetics can be induced by addition of salt^6^ or by a change in the peptide concentration.^11^ Recently, Hasecke et al. observed the transition from the nucleated to downhill aggregation kinetics for Aβ dimers and the hewL protein.^12^ They concluded that at low protein concentrations, most proteins aggregate according to sigmoidal kinetics with a clearly visible lag phase, whereas at high concentrations, the lag phase disappears due to rapid oligomer formation. Those small oligomers, both on- and off-pathway, can be more toxic than the mature fibril.^12^ Examination of the formation and transformation of transient oligomers is difficult for experimental studies due to the short lifetimes of transient states.^11^ The small oligomers can be measured only indirectly in most cases.

Computer simulation can support experimental studies by giving an insight at the molecular level. Coarse-grained models for protein aggregation are commonly used to expand the simulation time and system sizes.^13^ One type of coarse-grained models presents peptides as chains of superatoms where each peptide residue is mapped to one superatom.^14−16^ Interestingly, such simple models can reproduce a variety of equilibrium structures observed in experiment. In a series of papers, Janke and colleagues studied the thermodynamics of peptide aggregation using a homopolymer model.^14,17,18^ Ranganathan et al. connected the interaction strength, bending stiffness, and polymer chain length with various signatures of protein aggregation and amyloid formation.^15^ This leads to a hypothesis that the stiffness of polymer chains and interchain interactions are the distinguishing parameters for peptide equilibrium aggregate morphologies.^15,16^ Here, we extend this hypothesis to non-equilibrium aggregation and argue that the chain stiffness and interchain interactions also control the kinetics: from no observed aggregation to nucleated and downhill aggregation.

In this work, we study the peptide aggregation kinetics using a coarse-grained implicit solvent model introduced in our previous work.^16^ Our model presents peptides as strings of superatoms. Such model may represent intrinsically disordered homopetides: polyalanine, polyasparagine, or polyglutamine. On the other hand, as no explicit side chains are present, it may be viewed as a model that brings out the role of backbone interactions, which is consistent with the observation that the intermolecular backbone–backbone interactions may be a main factor responsible for the structure of a mature fibril and its aggregation propensity.^19^ By scanning the peptide–peptide interaction strength and chain stiffness, we recovered different kinetic behaviors observed in experiment, including nucleated and downhill aggregation. We address three major questions. First, what is the origin of the crossover from no-aggregation to nucleated and downhill aggregation? We argue that the interchain attraction is the primary quantity determining the aggregation propensity, whereas the stiffness is a secondary parameter. Second, are the aggregation kinetics correlated with the transient aggregate morphologies? Our simulations show that the aggregation rates and transient morphologies are determined by both the interchain attraction and intrachain bending stiffness: more attractive and stiff chains aggregate more rapidly and their transient morphologies are more ordered. Third, what is the effect of the chain length on the aggregation kinetics and morphologies? We found that longer chains have a larger aggregation propensity and aggregate faster, forming more regular aggregates.

## 2 Methods

Our coarse-grained implicit solvent model was described in detail in our previous work.^16^

**Figure 1:**
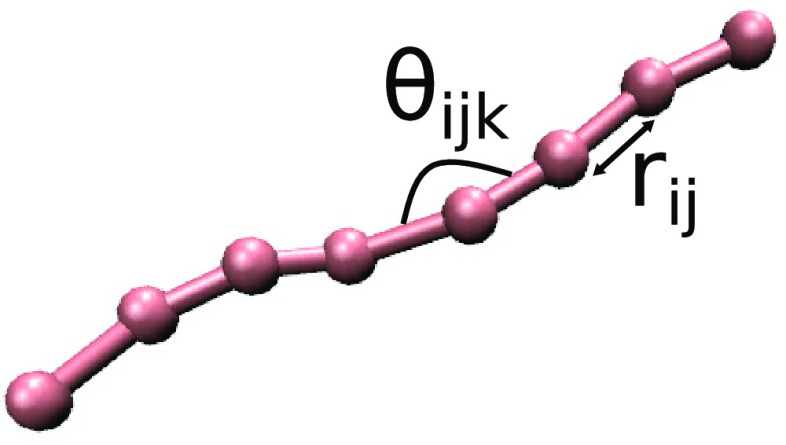
Homopeptide with 8 superatoms (SA8), with one superatom per residue. Bonded interactions are described by the bond length *r*_*ij*_ (eq 1) and the angle θ_*ijk*_ (eq 2).

Peptides are chains of Lennard-Jones superatoms representing residues (Figure 1) bonded via the harmonic bond potential
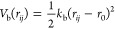
1where *i*, *j* represent two consecutive superatoms, *k*_b_ is the force constant for this study (fixed at 1250 kJ/(mol nm^2^)), and *r*_0_ is the equilibrium bond length (fixed at 0.35 nm).

The stiffness of the chain is determined by the cosine-based angle potential
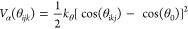
2where *i*, *j*, *k* represent three consecutive superatoms and θ_0_ is the equilibrium bond angle fixed at 180°. The simulations were carried out for six values of the angle force constant *k*_θ_: 10, 100, 200, 400, 800, and 1000 kJ/mol.

The nonbonded interactions between superatoms are defined by the Lennard–Jones potential
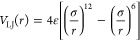
3where *r* is the distance between two nonbonded superatoms, ε is the depth of potential minimum, and σ is the distance at which LJ potential is equal to zero. In our simulations, σ is fixed at 0.47 nm and ε takes six values: 1.3, 1.4, 1.5, 1.6, 1.7, and 2.0 kJ/mol.

In this work, we vary the chain stiffness, *k*_θ_, and the strength of the peptide–peptide interactions, ε, to sample different peptide aggregation behaviors. Our focus is on the aggregation kinetics (in particular, on nucleated vs downhill aggregation).

The GROMACS 4.6.5 package^20^ was used for all simulations. The dynamics was propagated with a leap-frog stochastic dynamics integrator, which also serves as a thermostat at 303 K, and with periodic boundary conditions in all directions. The integration time step was 25 fs. With that time step our simulations were stable for a wide range of molecular parameters studied here. A similar time step, 30-40 fs, was used in the dry MARTINI force field.^21^ All simulations were carried out with an implicit solvent, which is defined by the friction coefficient used with the stochastic dynamics integrator. To mimic the friction effect of the solvent, an inverse friction coefficient of 0.17 ps was applied. This value was chosen to match the diffusion coefficient of a single molecule with that for the MARTINI model.^22^ MARTINI is often used to study peptide aggregation.^23,24^ Moreover, the MARTINI representations of polyalanine and polyglycine are similar to our peptide model. For that reason, we choose the friction coefficient based on the MARTINI kinetics.

In our preliminary simulations, we identified the monomer concentration *c*_0_ and the ranges of ϵ and *k*_θ_ values that include no aggregation, nucleated aggregation, and downhill aggregation. We wanted to sample that region to identify the boundaries between the aggregation modes. The kinetic behavior of peptides built from 8 superatoms (SA8) was simulated for 36 combinations of ε and *k*_θ_. The number of selected (ϵ, *k*_θ_) pairs is a compromise between the precision of detecting the region boundaries and the limited computer resources. The simulation time was 10 μs in each case. The simulations started with random initial configurations and were repeated at least five times to reduce the statistical noise. The initial concentration of the monomers was *c*_0_ = 2.8 mM, which corresponds to the superatom concentration *c*_SA_ = 22.3 mM. For a selected system showing nucleated aggregation, the simulations were repeated 25 times to quantify the critical cluster size and the average lag time.

To study the effect of the initial monomer concentration, we complemented the simulations for ε = 1.6 kJ/mol, *k*_θ_ = 200 kJ/mol, and *c*_0_ = 2.8 mM with simulations at lower concentrations, *c*_0_ = 1.4 and 0.7 mM.

To study the effect of the chain length, we also simulated the aggregation kinetics of peptides built from 16 superatoms (SA16). To allow a fair comparison with SA8 peptides, we kept the same superatom concentration, *c*_SA_ = 22.3 mM.

When reporting on repeated simulations, we show the standard deviation of the mean for the kinetic curves and the standard deviation of the sample for the averaged structural parameters as functions of the cluster size, *M*. The standard deviation of the sample better represents the data scatter when non-equilibrium trajectories do not sample all cluster sizes, *M*. Note that our simulations concern the initial, non-equilibrium phase of peptide aggregation.

The definition of a cluster was based on a cutoff distance: a peptide belongs to a cluster if the distance between an atom of this peptide and an atom of a different peptide in the cluster is equal to or less than 5.5 Å. The cutoff value 5.5 Å was chosen from the first maximum on the distribution of atom distances in clusters.^16^

The structural features of the aggregates were described by several descriptors: the end-to-end correlation parameter *C***_n_**, the radius of gyration *R*_g_, and the asphericity *b*. The end-to-end correlation parameter, *C***_n_**, was introduced in the polymer physics literature to characterize the aggregation transition of polymer systems.^14^ It is defined as
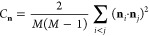
4where the unit vector **n**_*i*_ is the normalized end-to-end vector of the backbone atoms of peptide *i*. The parameter *C*_**n**_ describes the order of polymer chains in an aggregate and takes a value of 1 for the parallel alignment of chains and a value of around 0.3 for the random orientation. The asphericity *b* is defined as *b* = λ_*z*_^2^ – (λ_*x*_^2^ + λ_*y*_^2^)/2, where λ_*x*_, λ_*y*_, and λ_*z*_ are the principal moments of the gyration tensor and the axes are chosen such that λ_*x*_^2^ ≤ λ_*y*_^2^ ≤ λ_*z*_^2^.

The aggregation kinetics are investigated by following the number of free monomers, *N*_m_, and the cluster sizes, *M*, including the size of the largest aggregate, *M*_max_. To shorten the notation, we use the symbol *m* for the size of the largest cluster, *m* = *M*_max_ (eq 6).

To compare the different kinetic curves, the number of monomers is scaled as
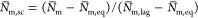
5where *N̅*_m,eq_ is the average number of monomers in the equilibrium phase and *N̅*_m,lag_ is the average number of monomers in the lag phase. The average is taken over the simulation repeats. For each nucleated aggregation repeat, the time is first shifted by the nucleation time, t*. Finally, the (shifted) time is scaled by the half-time, *t*_1/2_, i.e., the time when the monomers drop to *N̅*_m_ = 1/2 *N̅*_m,lag_. When the scaled curves overlap, we take this as an indication that the underlying molecular mechanisms are similar.

**Figure 2:**
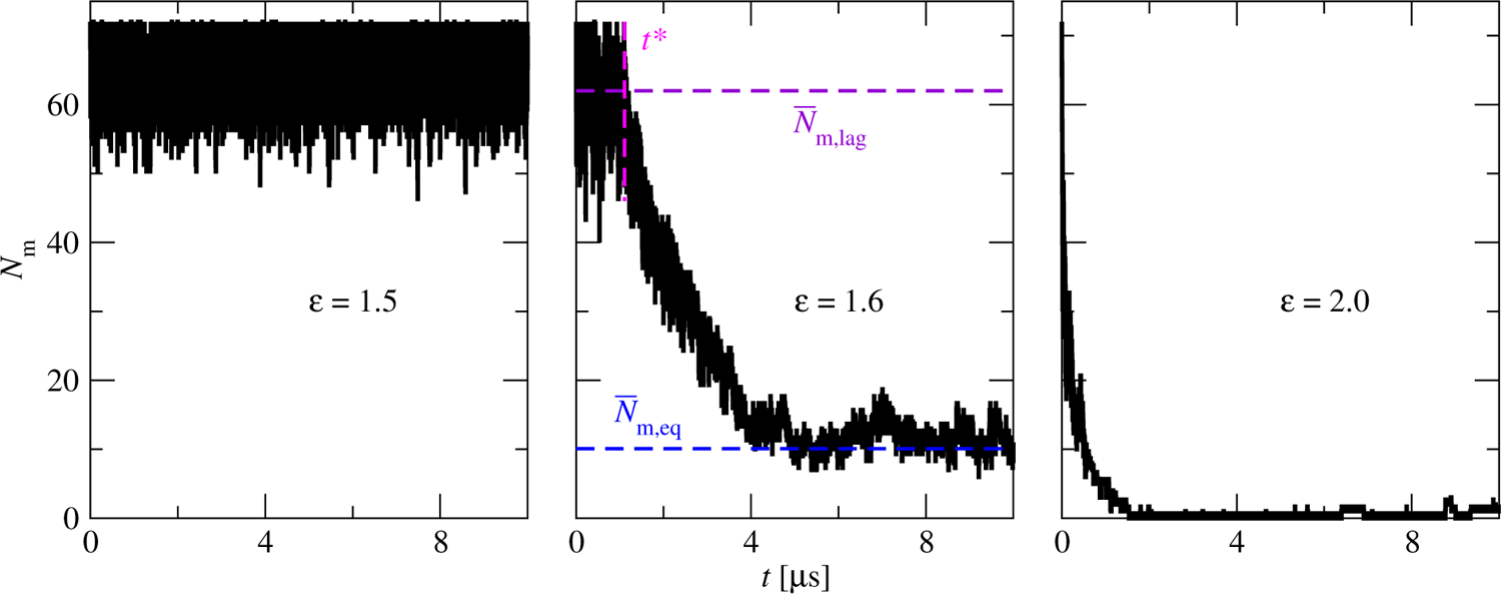
Examples of monomer kinetic curves for the three kinetic modes: no-aggregation (left panel), nucleated aggregation (middle panel), and downhill aggregation (right panel). The stiffness *k*_θ_ = 10 kJ/mol and the interaction strengths ε = 1.5, 1.6, and 2.0 kJ/mol are as indicated. Also indicated are the average number of monomers in the equilibrium phase *N̅*_m, eq_, the average number of monomers in the lag phase *N̅*_m, lag_, and the nucleation time, t*.

The nucleation time, t*, is defined here as the duration of the lag phase on a monomer kinetics curve (Figure 2). The nucleation time is a random quantity. The average nucleation time is denoted as τ*. To estimate the nucleation time, we use a change point (CP) analysis: a linear regression is applied to the consecutive monomer trajectory segments and a large change of the slope indicates the end of the lag phase. The average nucleation time estimated by the change point analysis is denoted as τ_CP_^*^.

The average nucleation time, τ*, together with the critical nucleus size, m*, can also be determined from the mean first-passage times (MFPT) as proposed by Wedekind et al.^25^ We use an extension of this method by Yi et al.^26^ In the MFPT method, the average first-arrival time of cluster size *m* is approximated as
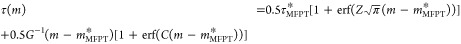
6where *m*_MFPT_^*^ is the critical nucleus size, *G* is the growth rate, *Z* is the Zeldovich factor, and τ*_MFPT_ is the average nucleation time.

For aggregating systems, the critical nucleus size, m*, was also estimated from the transition probability matrix (TPM). The transition probability matrix gives the probabilities of changes of the largest cluster size, *m* = *M*_max_. To estimate the transition probability matrix, the transitions between different states are counted and then normalized. The growth probability, *P*_growth_(*m*), of an aggregate of size *m* is defined as *P*_growth_(*m*) = *P*_f_(*m*) – *P*_b_(*m*), where the forward transition probability *P*_f_(*m*) is the transition probability from state *m* to *m* + 1 and the backward transition probability *P*_b_(*m*) is the transition probability from state *m* to *m* – 1. The critical nucleus size, *m*_TPM_^*^, is defined as the smallest aggregate size with *P*_growth_(*m*) ≥ 0. Note that the critical nucleus size based on the MFPT method is denoted as *m*_MFPT_^*^. Examples of the critical nucleus size analysis are presented in Supporting Information, see Figure S1.

Some nucleating systems do not aggregate in all simulation repeats. We report on such a system by listing the number of repeats with nucleation out of the total number of simulations (Table 1).

**Table 1:** Critical Nucleus Sizes, m*, and the Average Nucleation Times, τ*, for Different ε and *k*_θ_ values in kJ/mol (Compare the Phase Diagram in Figure 3)^a^

	ε = 1.4	ε = 1.4	ε = 1.5	ε = 1.5	ε = 1.6
	*k*_θ_ = 400	*k*_θ_ = 1000	*k*_θ_ = 100	*k*_θ_ = 400	*k*_θ_ = 10
*m*_TPM_^*^	12	8	7	8	10
*m*_MFPT_^*^	7.84	8.12	6.87	5.74	9.72
τ_CP_^*^ [ns]	τ_CP,10/25_^*^ = 3468 ± 3120	2730 ± 2510	640 ± 544	245 ± 265	τ_CP,20/25_^*^ = 3269 ± 2841
τ_MFTP_^*^ [ns]	4156	2688	706	268	3486

a The analysis methods are indicated as subscripts: CP - the change point analysis, MFTP - the mean first-passage time method, and TPM - the transition probability matrix approach. If aggregation did not occur in all 25 simulation repeats, the number of repeats showing aggregation, e.g., 10, is also noted in the subscript, e.g., 10/25.

## 3 Results and Discussion

### 3.1 Crossover from No-aggregation to Nucleated Aggregation and Downhill Aggregation

Our simulations indicate that the aggregation kinetics are strongly dependent on the interaction strength, ε, and chain stiffness, *k*_θ_. Figure 2 shows examples of the three aggregation modes observed in our simulations: no-aggregation for small ε and *k*_θ_, where only small, unstable aggregates are observed; downhill aggregation for high ε and *k*_θ_, where monomers decay rapidly and no lag phase is observed; and nucleated aggregation for intermediate ε and *k*_θ_, where a lag phase is seen. See also the movies in Supporting Information, illustrating the three types of kinetic behavior.

**Figure 3:**
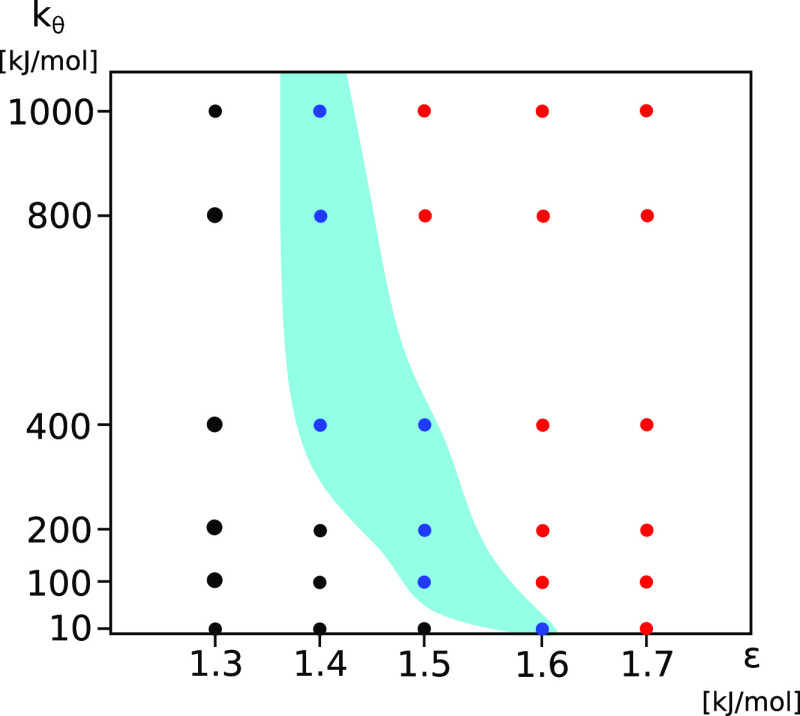
Aggregation kinetics phase diagram for different interaction strengths, ε, and chain stiffnesses, *k*_θ_. The color of the points indicates three different aggregation behaviors. The blue points denote nucleated aggregation, red ones denote downhill aggregation, and black ones denote no-aggregation systems. The blue area is added for better visualization of the nucleated aggregation region.

Figure 3 shows a kinetic phase diagram indicating the dependence of the aggregation mode on ε and *k*_θ_. The interaction strength, ε, is the primary parameter determining the observed aggregation mode: for each *k*_θ_, the aggregation mode can be changed by increasing the ε. The stiffness, *k*_θ_, is a secondary parameter as it does not change the aggregation mode for small (no aggregation) and high (downhill aggregation) strengths, ε. The observed aggregation mode is sensitive to *k*_θ_ only for intermediate values of ε. Note, however, that the aggregation rates and transient morphologies are determined by both ε and *k*_θ_: for higher ε and *k*_θ_, the aggregation is faster and the aggregate structure is more regular (see below).

Because of the simulation time limitations, it is difficult to determine precisely the borderlines between the no-aggregation, nucleated aggregation, and downhill aggregation regions in the (ϵ, κ) phase plane. For instance, a system where no aggregation is observed may just have a long lag time. Similarly, some nucleating systems, e.g., ε = 1.4 and *k* = 400, do not aggregate in all simulation repeats.

**Figure 4:**
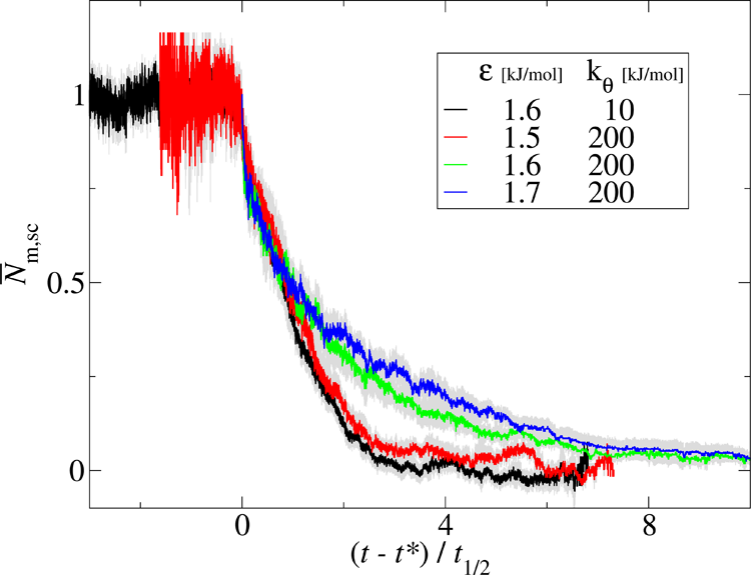
Scaled number of free monomers, *N̅*_m,sc_ (eq 5), as a function of time for four systems: ε = 1.6 kJ/mol, *k*_θ_ = 10 kJ/mol (black line); ε = 1.5 kJ/mol, *k*_θ_ = 200 kJ/mol (red line); ε = 1.6 kJ/mol, *k*_θ_ = 200 kJ/mol (green line); and ε = 1.7 kJ/mol, *k*_θ_ = 200 kJ/mol (blue line). Time is first shifted by the nucleation time, t*, and then scaled by the half-time, *t*_1/2_. For each curve, the gray area shows the standard deviation of the mean.

In order to compare the nucleated and downhill aggregation kinetics, the monomer kinetics curves are presented in Figure 4 for four systems: ε = 1.6 kJ/mol, *k*_θ_ = 10 kJ/mol (black line); ε = 1.5 kJ/mol, *k*_θ_ = 200 kJ/mol (red line); ε = 1.6 kJ/mol, *k*_θ_ = 200 kJ/mol (green line); and ε = 1.7 kJ/mol, *k*_θ_ = 200 kJ/mol (blue line). The scaled kinetics curves for two systems, i.e., ε = 1.6 kJ/mol, *k*_θ_ = 10 kJ/mol (black line); and ε = 1.5 kJ/mol, *k*_θ_ = 200 kJ/mol (red line), have a visible lag (nucleation) phase, whereas for two other systems, i.e., ε = 1.6, *k*_θ_ = 200 (green line); and ε = 1.7 kJ/mol, *k*_θ_ = 200 kJ/mol (blue line), nucleation is not observed. The scaled curves in Figure 4 emphasize the difference between the postnucleation and downhill aggregation kinetics.

**Figure 5:**
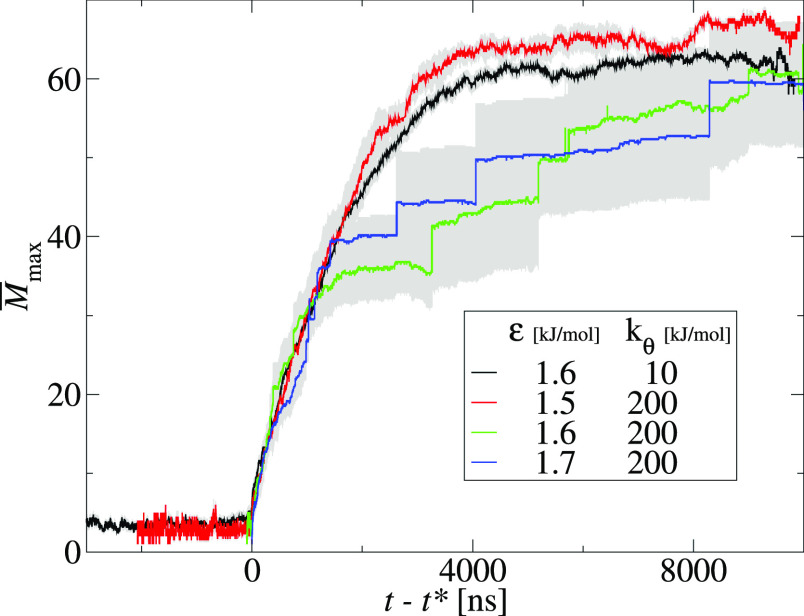
Average size of the largest cluster, *M̅*_max_, as a function of time for two nucleated aggregation systems: ε = 1.6 kJ/mol, *k*_θ_ = 10 kJ/mol (black line); and ε = 1.5 kJ/mol and *k*_θ_ = 200 kJ/mol (red line); and two downhill aggregation systems: ε = 1.6 kJ/mol, *k*_θ_ = 200 kJ/mol (green line); and ε = 1.7 kJ/mol, *k*_θ_ = 200 kJ/mol (blue line). Time is shifted by the nucleation time, t*. For each curve, the gray area shows the standard deviation of the mean.

The difference in the postnucleation and downhill aggregation kinetics is also visible for the largest cluster growth. Figure 5 presents the average largest cluster size, *M̅*_max_, as a function of time shifted by the nucleation time, *t* – t*, for the same four systems as in Figure 4. Initially, the largest cluster grows by monomer addition. For the nucleated aggregation, monomer addition remains the dominant path of cluster growth up to the end of simulation. On the other hand, for downhill aggregation, the cluster–cluster coalescence events, seen as jumps of the kinetic curves, contribute to the largest cluster growth after the first 1000 ns. Note that no system reached the maximum aggregate size, *M* = 72. For the nucleated aggregation, at the end of the simulations, free monomers coexist with one large cluster, whereas for downhill aggregation, more than one aggregate is present at the final stage.

For systems showing nucleated aggregation, we attempted to estimate the average nucleation time, τ*, and critical cluster size, m* (Table 1). The average nucleation time, τ*, decreases with increasing *k*_θ_ for the same ε, and with increasing ε for the same *k*_θ_. For the critical nucleus size, we do not see such clear correlations. Thus, we conclude that, for our model, the critical nucleus size is about 10 but its dependence on ε and *k*_θ_ remains unresolved.

Several coarse-grained models have been applied to study the peptide nucleation pathways.^27−32^ For a given molecular system, the critical nucleus size, n*, depends on the initial concentration. For instance, n* for polyglutamine was estimated by Haaga et al. as ranging from n* = 4 to 12, depending on the supersaturation.^32^ Notably, Saric et al.^28^ compared the nucleation of their patchy spherocylinder model for low and high β-propensity proteins. They found that the average aggregation number of the nucleating oligomer changes between n* ≈ 2–12 for the low β-propensity proteins and between n* ≈ 2–4 for the high β-propensity proteins. In our model, we do not see a clear dependence of the nucleus size on the system-determining parameters, *k*_θ_ and ε. This may related to the difference in the model dynamics. In the spherocylinder model, the low and high aggregation propensity states are explicitly built into the model, whereas in the present model, the chain fluctuations are controlled by the chain stiffness and the attraction strength. However, we cannot exclude the possibility that the increased sampling might reveal trends similar to those for the spherocylinder model.

**Figure 6:**
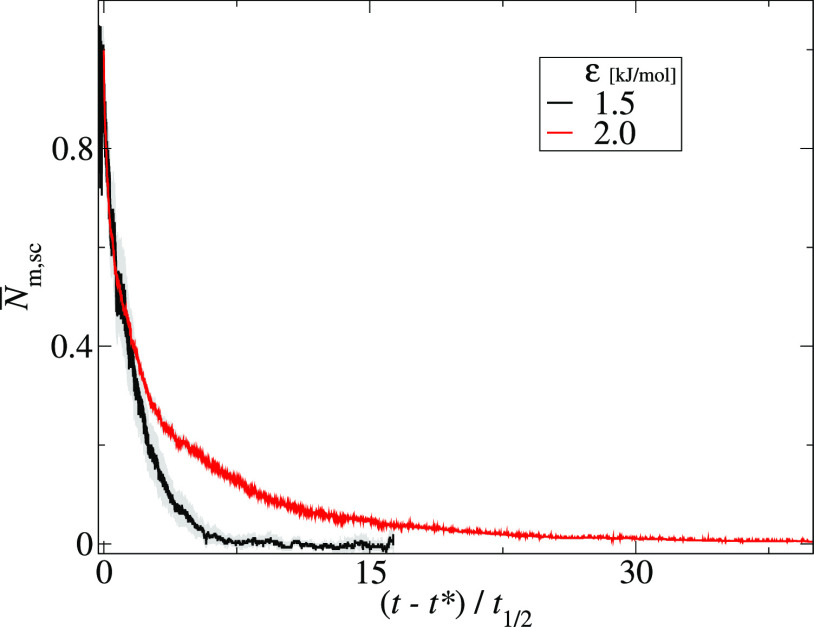
Trajectory of the scaled number of monomers, *N̅*_m,sc_ (eq 5) for two systems with the same chain stiffness *k* = 1000 kJ/mol, and various interaction strengths, ε = 1.5 kJ/mol (black line) and 2.0 kJ/mol (red line). For each curve, the gray area shows the standard deviation of the mean.

For downhill aggregation, different monomers kinetics are observed. For strong interactions between peptides, ε ≥ 2.0 kJ/mol, the monomers decay almost to zero and disaggregation events are rarely observed. On the other hand, for weaker interactions, ε ≤ 2.0, the aggregates coexist with monomers till the end of the simulation. It is worth noting that the scaled monomer kinetics curves for weak and strong interactions do not overlap (Figure 6).

### 3.2 Aggregation Kinetics vs Transient Aggregate Morphologies

**Figure 7:**
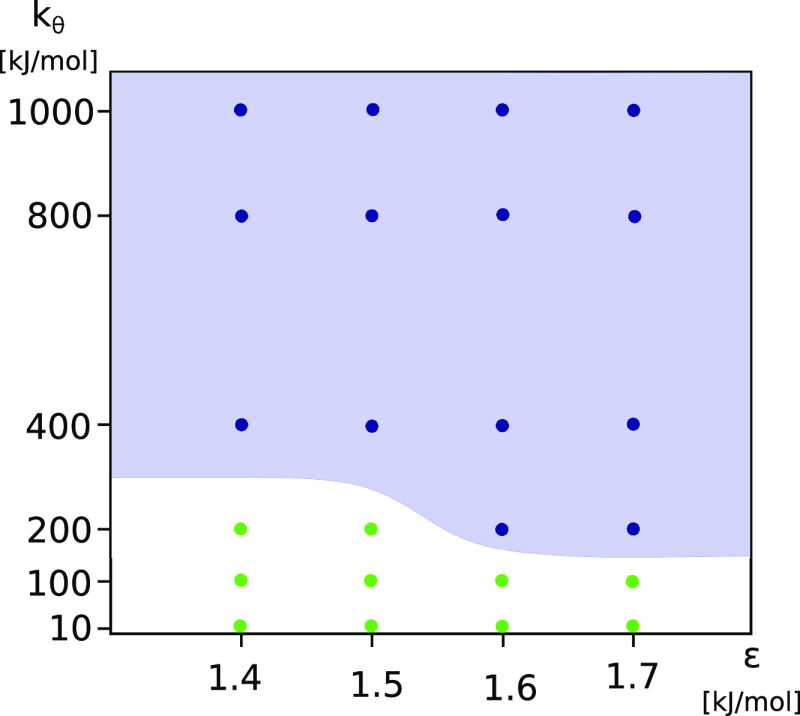
Structural phase diagram for different interaction strengths, ε, and chain stiffnesses, *k*_θ_. The color of points indicates the two different aggregate structures. The blue points denote ordered aggregates with parallel peptide alignment and green ones disordered clusters. The blue area is added for better visualization of the ordered region.

In aggregating systems, an increase of the chain stiffness changes the aggregate structures from amorphous (spherical and disordered) to structured ones with parallel peptide arrangement (Figure 7). This effect can be investigated by following the structural parameters as functions of the aggregate size.

**Figure 8:**
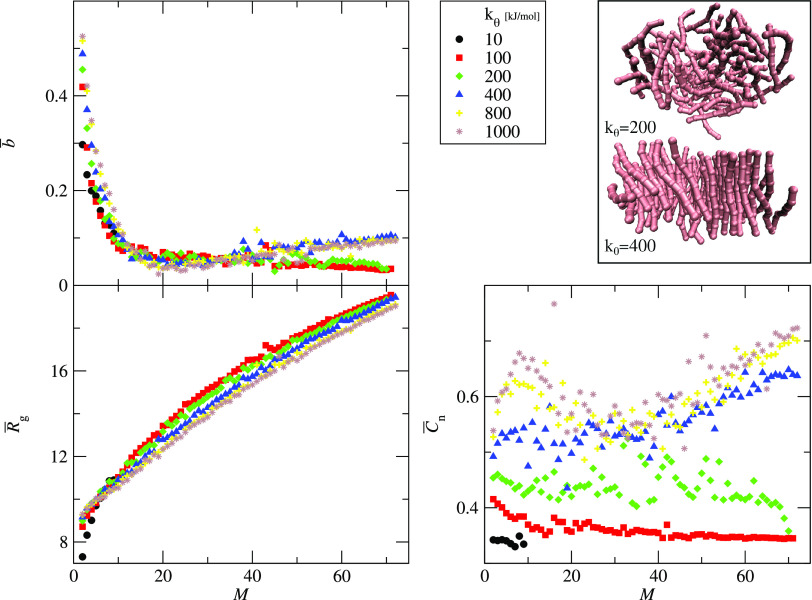
Three structural parameters as functions of the aggregate size, *M*: the average asphericity, *b̅* (upper left panel), average radius of gyration, *R̅*_g_ (bottom left panel), and average end-to-end correlation parameter, *C̅***_n_** (bottom right panel). The data is presented for systems with variable chain stiffness, 10 ≤ *k*_θ_ ≤ 1000 kJ/mol, as indicated. The interaction strength is constant, ε = 1.5 kJ/mol. The right upper panel shows the sample structures of the final aggregates for two chain stiffnesses, *k*_θ_ = 200 kJ/mol (upper) and *k*_θ_ = 400 kJ/mol (bottom). For clarity, only the average values are shown. Figure S2 in Supporting Information shows also the error bars representing the standard deviation of the sample.

A way of comparing the time evolution of the structural parameters is to plot them as conditional averages of the aggregate size, *M*. For instance, from the aggregation trajectories, one can extract the cluster asphericity–cluster size data, (*b*, *M*), and take the average over all frames in all repeats to obtain a plot of the average asphericity *b̅* vs the cluster size, *M*. Due to the limited number of simulation repeats, a cluster of a given size *M* can be formed by transient aggregation in a single repeat. For this reason, we report the error bars as the standard deviation of the sample. Figure 8 shows three structural parameters: the average asphericity, *b̅*, average radius of gyration, *R̅*_g_, and average end-to-end correlation parameter, *C̅***_n_**, as functions of the aggregate size, *M*, for ε = 1.5 kJ/mol and *k*_θ_ = 10 – 1000 kJ/mol. For small aggregates, *M* < 20, the asphericity decreases for all systems. For flexible chains, *k*_θ_ < 400 kJ/mol, the asphericity still decreases but with a smaller slope. On the other hand, for rigid peptides, *k*_θ_ ≥ 400 kJ/mol, the average asphericity increases for large aggregates, *M* > 20. These two types of asphericity behavior are connected with the aggregate structures presented in the right upper panel of Figure 8. The top structure is an example of the final aggregate formed by a peptide with low chain stiffness, *k*_θ_ = 200, and it is mostly spherical and disordered, whereas the bottom structure corresponds to the peptides with higher chain stiffness, *k*_θ_ = 400. This cluster has a parallel arrangement of peptide chains forming a single-layer structure. This arrangement is supported by the end-to-end correlation parameter, which is significantly greater for stiffer peptides, *k*_θ_ ≥ 400 kJ/mol. Especially, this difference is visible for larger aggregates, *M* ≥ 40. Also, the average radius of gyration shows different shapes of curves for each *k*_θ_. The curvature is the largest for small *k*_θ_ (see *k*_θ_ ≤ 200 in Figure 8). For rigid chains, *R̅*_g_ vs *M* is almost linear (see *k*_θ_ ≥ 800 kJ/mol in Figure 8).

**Figure 9:**
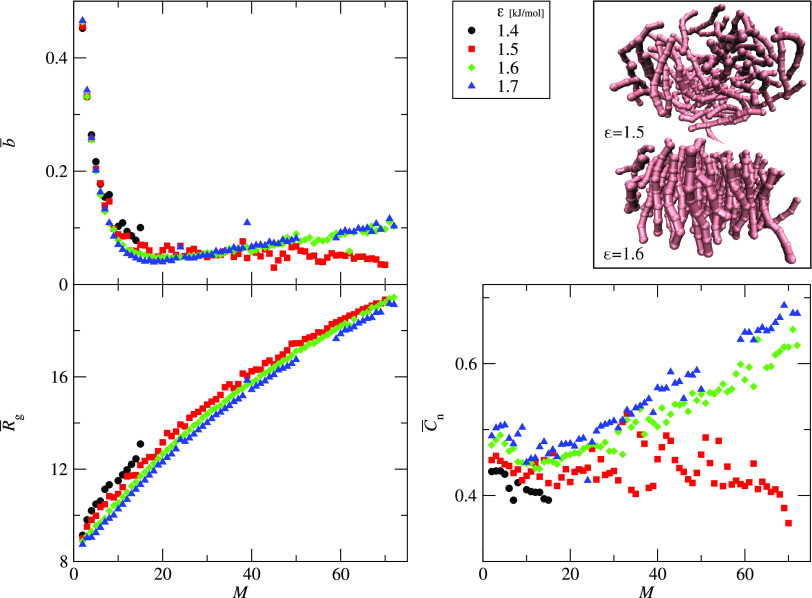
Three structural parameters as functions of the aggregate size, *M*: the average asphericity, *b̅* (upper left panel), average radius of gyration, *R̅*_g_ (bottom left panel), and average end-to-end correlation parameter, *C̅***_n_** (bottom right panel). The data is presented for systems with the variable interaction strength, 1.4 ≤ ε ≤ 1.7 kJ/mol, as indicated. The chain stiffness is constant, *k*_θ_ = 200 kJ/mol. The right upper panel shows the sample structure of final aggregates for ε = 1.5 kJ/mol (upper) and ε = 1.6 kJ/mol (bottom). For clarity, only the average values are shown. Figure S3 in Supporting Information shows also the error bars representing the standard deviation of the sample.

The aggregate structures also change with interaction strength at a constant chain stiffness. Figure 9 shows three structural parameters: average asphericity, *b̅*, average radius of gyration, *R̅*_g_, and average end-to-end correlation parameter, *C̅***_n_**, as functions of the aggregate size for various interaction strengths, 1.4 ≥ ε ≥ 1.7 kJ/mol, at the same chain stiffness, *k*_θ_ = 200 kJ/mol. The structural differences are clearly visible for the average asphericity, *b̅*. Two aggregation types can be distinguished. For low interaction strengths, ε ≥ 1.5 kJ/mol, aggregation follows the isotropic, three-dimensional (3D) mechanism and leads to spherical aggregates (see the top structure in Figure 9 right upper panel). For higher interaction strengths, 1.6 ≥ ε kJ/mol, aggregation leads to the formation of single-layer-like aggregates (see the bottom structure in Figure 9 right upper panel), which is connected with the growth of asphericity, *b̅*. The average end-to-end correlation parameter, *C̅***_n_**, supports this division. The spherical aggregates formed by weakly interacting peptides, ε ≥ 1.5 kJ/mol, do not show internal order and *C̅***_n_** stays close to 0.33 for all cluster sizes. On the other hand, two-dimensional (2D) aggregates formed by peptides with ε ≥ 1.6 kJ/mol have the parallel peptide arrangement shown as the growth of *C̅***_n_**. The trajectories of the average radius of gyration, *R̅*_g_, have quite similar shapes for all systems. However, the curves show the transition to smaller values with increasing interaction strengths. This behavior indicates that, independent of the aggregate structure, the peptides are denser for higher ε. This structurally different behavior does not induce a change in the monomers’ attachment–detachment kinetics. It suggests that the peptide reorientation is faster than the monomer addition.

We found that more rigid peptides aggregate faster and form more ordered aggregates than the flexible ones with the same interaction strength. It can be explained by considering the monomer conformations. Flexible chains can collapse to minimize the peptide-environment surface and the monomer collapse competes with peptide aggregation. On the other hand, for rigid peptides, the aggregation process is the only way to reduce the peptide-environment surface. We speculate that the conditions increasing the peptide or protein stiffness will increase the aggregation propensity. One example is phosphorylation, which leads to an increase in the persistence length of peptide chains.^33^ Interestingly, the phosphorylated proteins show a stronger tendency to aggregate than the unphosphorylated ones.^34,35^ The correlation between the persistence length and the aggregation propensity was found also for other peptides and proteins. Yan and Wang found that Aβ42 has a more rigid C-terminus than Aβ40.^36^ Aβ42 is more prone to aggregation than Aβ40. The more rigid terminus can support a β-conformation, so it can be interpreted as an internal pre-nucleus for fibril formation. Another example is polyglutamine. Singh and Lapidus suggested that the increased stiffness of polyglutamine chains is responsible for the aggregation propensity.^37^

It is worth noting that in our simulations, the transition from disordered to ordered aggregates is sharp: we do not observe the coexistence of aggregates with various morphologies (ordered vs amorphous) or the reorganization from the disordered to ordered aggregates. When the interaction strength or chain stiffness is higher than a critical value, cylindrical aggregates with parallel peptide alignment are formed. The peptide alignment becomes more regular with the further growth of ε and *k*_θ_, but the overall morphology stays similar.

### 3.3 Effect of the Concentration

**Figure 10:**
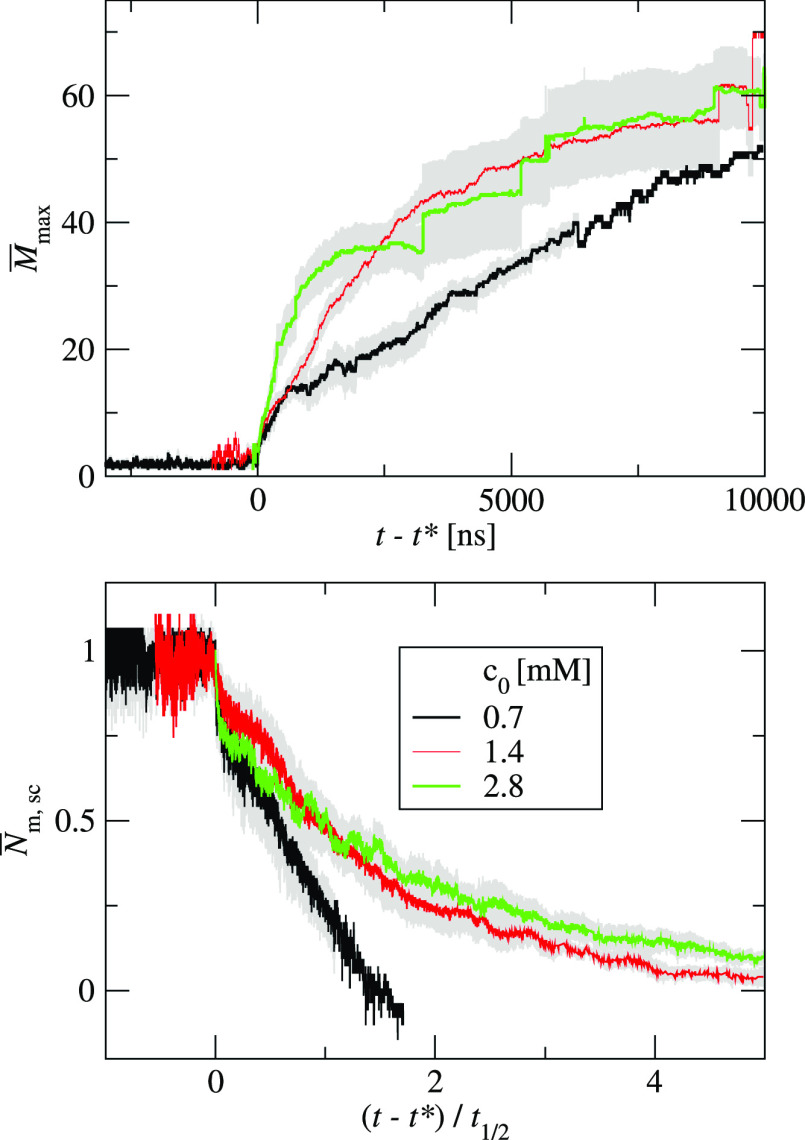
Kinetic curves for three initial concentrations: our standard concentration 2.8 mM (green lines), and two smaller concentrations: 1.4 mM (red lines) and 0.7 mM (black lines). The top panel shows the average size of the largest cluster, *M*_max_, as a function of time. The bottom panel shows the scaled number of monomers, *N̅*_m, sc_ (eq 5) as a function of the shifted and scaled time. For each curve, the gray area shows the standard deviation of the mean.

We studied the concentration effect for the system ε = 1.6 kJ/mol, *k*_θ_ = 200 kJ/mol. As expected, the aggregation propensity decreases with decreasing concentration (Figure 10). At our standard concentration, 2.8 mM, we observed downhill aggregation for this system. As the concentration decreases to 1.4 mM, the downhill mode changes to nucleated aggregation. As the concentration further decreases from 1.4 to 0.7 mM, the nucleation phase becomes longer. The average nucleation time is given by τ_CP_^*^ = 359 ± 323 ns and τ_CP,3/5_^*^ = 2 501 ± 1 734 ns for concentrations of 1.4 and 0.7 mM, respectively. For the lowest concentration, 0.7 mM, aggregation does not occur in two repeats. It suggests that this concentration is close to the no-aggregation region. When the shifted and scaled time reaches the value 1.09 for the monomer kinetics and the shifted time reaches the values 50 500 ns for the largest cluster kinetics, the standard deviation is no longer well defined for the repeats at the lowest concentration, 0.7 mM. It is caused by the fact that only one simulation repeat reaches a long shifted time. The time shift depends on the nucleation phase length. In our case, one simulation repeat has a much shorter nucleation time than the other repeats. This concentration effect is in line with an experimental observation that the transition between downhill and nucleated kinetics may be concentration-dependent.^11,12^ The structural properties of the aggregates do not change in the studied concentration range.

### 3.4 Effect of the Peptide Chain Length

**Figure 11:**
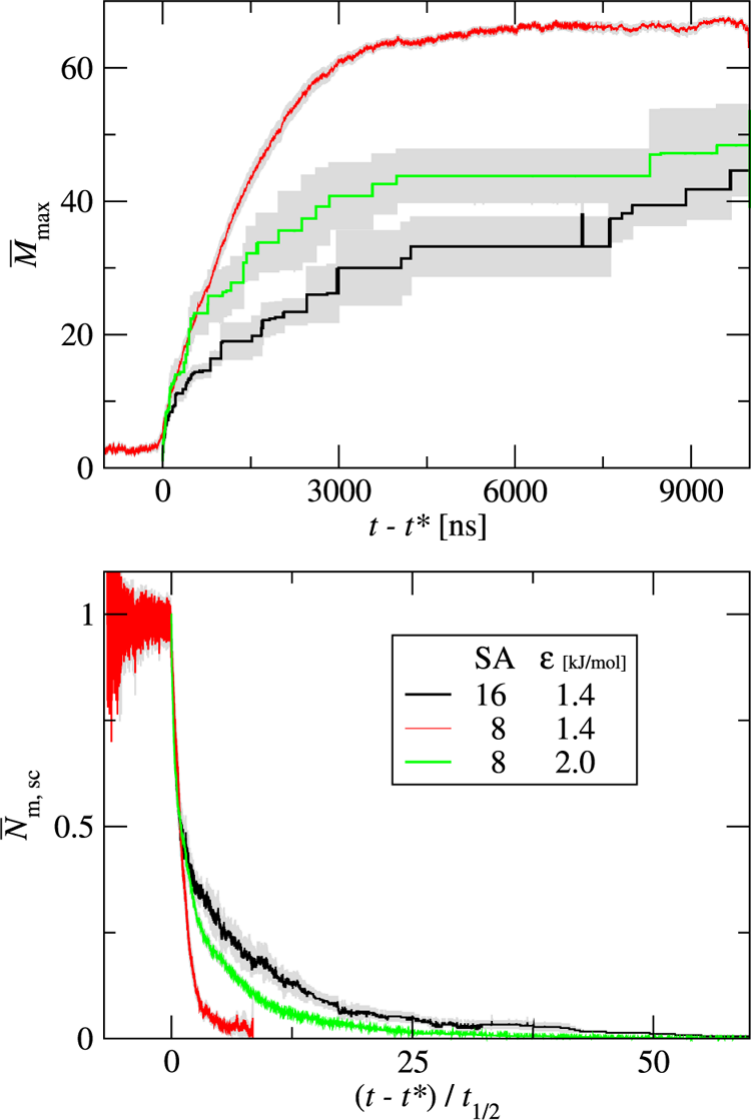
Kinetic curves for three systems: one for chains with 16 superatoms (SA16), with ε = 1.4 kJ/mol, *k*_θ_ = 1000 kJ/mol (black lines) and two for peptide chains with 8 superatoms (SA8), with *k*_θ_ = 1000 kJ/mol and various interaction strengths: ε = 1.4 kJ/mol (red lines) and ε = 2.0 kJ/mol (green lines). The top panel shows the average size of the largest cluster, *M*_max_, as a function of time. The bottom panel shows the scaled number of monomers, *N̅*_m, sc_ (eq 5) as a function of the shifted and scaled time. For each curve, the gray area shows the standard deviation of the mean.

We performed the simulation for peptides with 16 superatoms (SA16) to study the effect of chain length on aggregation. We kept constant the superatom concentration, *c*_SA_ = 22.3 mM. Figure 11 shows the kinetic curves for three systems: one for chains with 16 superatoms (SA16), with ε = 1.4 kJ/mol, *k*_θ_ = 1000 kJ/mol and two for peptide chains with 8 superatoms (SA8), with *k*_θ_ = 1000 kJ/mol and various interaction strengths: ε = 1.4 kJ/mol and ε = 2.0 kJ/mol. The bottom panel shows the scaled number of monomers, *N̅*_m,sc_, as a function of time shifted by nucleation time t* and scaled by the half-time, *t*_1/2_. The aggregation of long peptides (SA16, black lines) shows no nucleation phase. However, the short peptides (SA8) with the same interaction strength and chain stiffness aggregate with clearly nucleated kinetics (red lines). The short peptides with stronger interaction, ε = 2.0 (green lines), aggregate with similar kinetics as the longer peptides. For both short and long chains, the cluster–cluster coalescence contributes to the largest cluster growth.

Our simulations indicate that aggregation is faster for longer peptides. This can be explained as follows. The overall aggregation rate is the balance of aggregation and fragmentation events. For longer peptides, a monomer is locked within a cluster more strongly as it has more interaction sites. Thus, longer chains favor monomer association over monomer dissociation. This effect also stabilizes clusters against fragmentation. Moreover, the sticking probability of coagulating clusters is higher. This is further supported by the superatom concentration in monomers at equilibrium *c*_SA,m,eq_. This concentration is much lower for the long peptides (SA16), *c*_SA,m,eq_ = 1.8 × 10^–5^ mM, than for the short peptides (SA8), with the same ε, *c*_SA,m,eq_ = 1.2 mM, and also for the short peptides with a higher interaction strength (ε = 2.0 kJ/mol), *c*_SA,m,eq_ = 4.3 × 10^–4^ mM.

**Figure 12:**
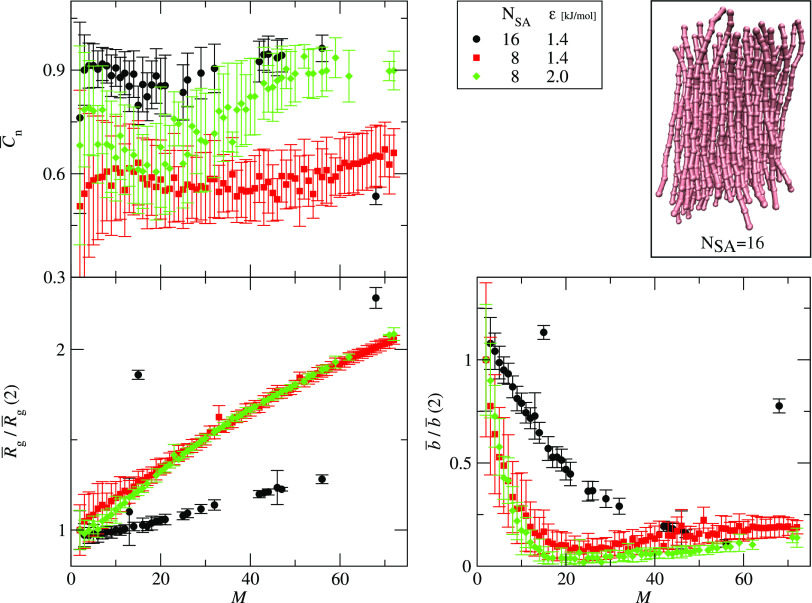
Three structural parameters as functions of the aggregate size, *M*: the average asphericity scaled by the dimer asphericity, *b̅*/ *b̅*(2) (bottom right panel), average radius of gyration scaled by the radius of gyration for dimers, *R̅*_g_/*R̅*_g_(2) (bottom left panel), and average end-to-end correlation parameter, *C̅***_n_** (upper left panel). The data is presented for three systems: one system with long, 16 superatom chains (SA16), with ε = 1.4, *k*_θ_ = 1000 (black circles), and two systems with short, 8 superatom chains (SA8), with *k*_θ_ = 1000 and the interaction strengths: ε = 1.4 (red squares) and ε = 2.0 (green diamonds).

The structural analysis for the same three systems shows more differences between aggregates formed by short peptides (SA8) and long peptides (SA16). The end-to-end correlation parameters, *C̅***_n_**, indicate that the aggregates formed by the long peptides are more ordered than the aggregates of short peptides. Also, the average radius of gyration and average asphericity normalized by their values for dimers are different for short and long peptides (Figure 12, bottom, left, and right panels, respectively). The growth of the average radius of gyration is slower for longer peptides than for the shorter ones. The average asphericity decreases initially for the three systems, but for the short peptides, the asphericity reaches a minimum and then increases, whereas for the long peptide the asphericity decreases for the entire range of aggregate sizes. This can be rationalized as follows. For rigid peptides, the aggregates have a cylindrical shape with the peptides parallel to the cylinder axis. When an aggregate grows, the height of the cylinder representing the aggregate stays roughly constant and equal to the length of peptide chains. However, the cylinder radius growths with the aggregate size, *M*. When the diameter of the cylinder base becomes comparable to the peptide length, the asphericity reaches a minimum. Thus, for longer peptides, the asphericity reaches a minimum for larger clusters.

On the other hand, the longer peptides also show nucleated kinetics for sufficiently low interaction strength as ε = 1.0 (Figures S4 and S5 in the Supporting Information). This suggests that, although the aggregation rate increases for longer peptides, the overall kinetic mechanism does not change.

The structural diagrams show a few outliners where the data points lie far away from other regularly changing values; see for instance the bottom left and right panels in Figure 12. These outliners correspond to rare events where a cluster of a given size *M* is formed by transient aggregation of two clusters and lasts for a few frames, say less than 2 ns. A typical restructuring time in our simulations is between 2 and 4 ns for large aggregates. When an unstable cluster has no time to rearrange into a stable configuration, its “unusual” structural properties are detected as outliners. Despite the presence of outliners, the size of an aggregate, *M*, seems to correlate well with the structural properties.

## 4 Conclusions and Outlook

Our simulations explored the hypothesis that the interplay between interchain attraction and intrachain bending stiffness controls the peptide aggregation kinetics and transient aggregate morphologies. We showed that our coarse-grained model of peptide aggregation reproduces the different kinetics behaviors observed in experiments. The peptide aggregation modes (no observed aggregation, nucleated, and downhill aggregation (Figure 2)) are mainly determined by the interchain interaction strength, but the peptide chain stiffness is also important for intermediate strengths (Figure 3).

The nucleated aggregation region of the kinetic phase diagram shows universal aggregation kinetics: the kinetic curves can be scaled to collapse onto one master curve (Figure 4). For the nucleated aggregation, the addition of monomers is the the main aggregation path (Figure 5). The average nucleation time decreases with increasing intrachain stiffness and interchain attraction (Table 1). However, we do not see such clear correlations for the critical nucleus size. In our model, the critical nucleus size is about 10 peptides but its dependence on the stiffness and interactions remains unresolved.

For the downhill aggregation, the scaled kinetic curves for low and high interactions do not overlap (Figure 6), which indicates a variable aggregation mechanism. For rapid, downhill aggregation, the cluster–cluster coalescence events are observed (Figure 11 top panel).

Both the interaction strengths and chain stiffness determine the aggregation rates and transient morphologies: the more attractive and stiff chains aggregate more rapidly and the aggregate structures are more ordered (Figures 8 and 9). Individual peptide molecules combine into clusters whose basic structures (amorphous or ordered) do not change as the clusters grow. Nevertheless, the growing clusters undergo internal reorganizations, i.e., become denser.

We found that, as the initial monomer concentration decreases, the downhill aggregation mode changes to nucleated aggregation (Figure 10). The structural properties of the aggregates do not change in the studied concentration range.

We found also that longer chains (16 superatoms vs 8 superatoms) have a larger aggregation propensity and aggregate faster, forming more regular aggregates (Figures 11 and 12). However, although the aggregation rate is larger for longer peptides, the overall kinetic mechanism does not change.

The present model suggests that the chain stiffness and molecular attraction are the determinants of the peptide aggregate morphologies and aggregation kinetics. However, this model does not lead to fibril-like structures. We note that fibrils were observed in simpler models that map the whole peptide chain as rod-like^31^ or spherocylinder^29^ particles. In these models, the peptide–peptide interactions are anistropic, e.g., only part of a particle is highly attractive. Such anisotropic interaction can mimic the effect of the side chains and the conformation fluctuations. Coarse-grained peptide models with an atomic resolution of one or more superatoms per residue can generate fibrillar structures when the molecular asymmetry is built-in at the residue level.^38−45^ For instance, a coarse-grained three-bead-per-residue model developed by Bellesia and Shea^40−42^ shows that the dihedral flexibility controls the aggregation kinetics and aggregate morphologies. A similar conclusion follows from a model developed by Caflisch and co-workers.^43−45^ However, as those models involve heterogeneous beads, side chains, and electrostatic charges, it is unclear whether the dihedral flexibility is the primary factor or other structural features are required for the fibrillization.

In our future work, we hope to identify the primary causes of fibrillization using a suitable modification of the present model. Specifically, we plan to study the effects of side chains and dihedral flexibility. Our preliminary simulations reveal, for instance, that the residue symmetry breaking by the presence of side chains can lead to the formation of short fibrils. When the end beads are different from those along the chain, one can see the formation of longer and more stable fibrils. From a more general perspective, we seek to develop a minimal coarse-grained residue-based peptide model that reproduces the broad spectrum of aggregate morphologies and aggregation kinetics and might be useful for developing and testing molecular theories of peptide aggregation.

## Supporting Information Available

Examples of the critical nucleus size determined by the TPM and MFPT methods, structural parameters as functions of the aggregate size for ε = 1.5, 10 ≤ *k*_θ_ ≤ 1000 with error bars, structural parameters as functions of the aggregate size for 1.4 ≤ ε ≤ 1.7, *k*_θ_ = 200 with error bars, examples of the monomer and cluster kinetic curves for the long peptide (SA16), examples of the structural analysis for the long peptide (SA16), movies illustrating the three type of kinetic behavior (PDF)
No-aggregation (MPG)
Nucleated aggregation (MPG)
Downhill aggregation (MPG)

## Supplementary Material

jp1c00290_si_001.pdf

jp1c00290_si_002.mpg

jp1c00290_si_003.mpg

jp1c00290_si_004.mpg
