# Supplementary material for: Diverse Aggregation Kinetics Predicted by a Coarse-Grained Peptide Model
Source: J Phys Chem B. 2021 Jul 12;125(28):7587–97. doi: 10.1021/acs.jpcb.1c00290 (PMC8389928; doi:10.1021/acs.jpcb.1c00290)
Supplement: Supplementary file 1 — jp1c00290_si_001.pdf [file jp1c00290_si_001.pdf]

# Supporting Information

for

Diverse Aggregation Kinetics Predicted by a Coarse-Grained Peptide Model

Beata Szała-Mendyk,† Andrzej Molski‡

Adam Mickiewicz University in Poznań, Faculty of Chemistry,

Umultowska 89b, 61-614 Poznań, Poland

†beata.szala@amu.edu.pl, ‡amolski@amu.edu.pl

## S1 Critical nucleus size

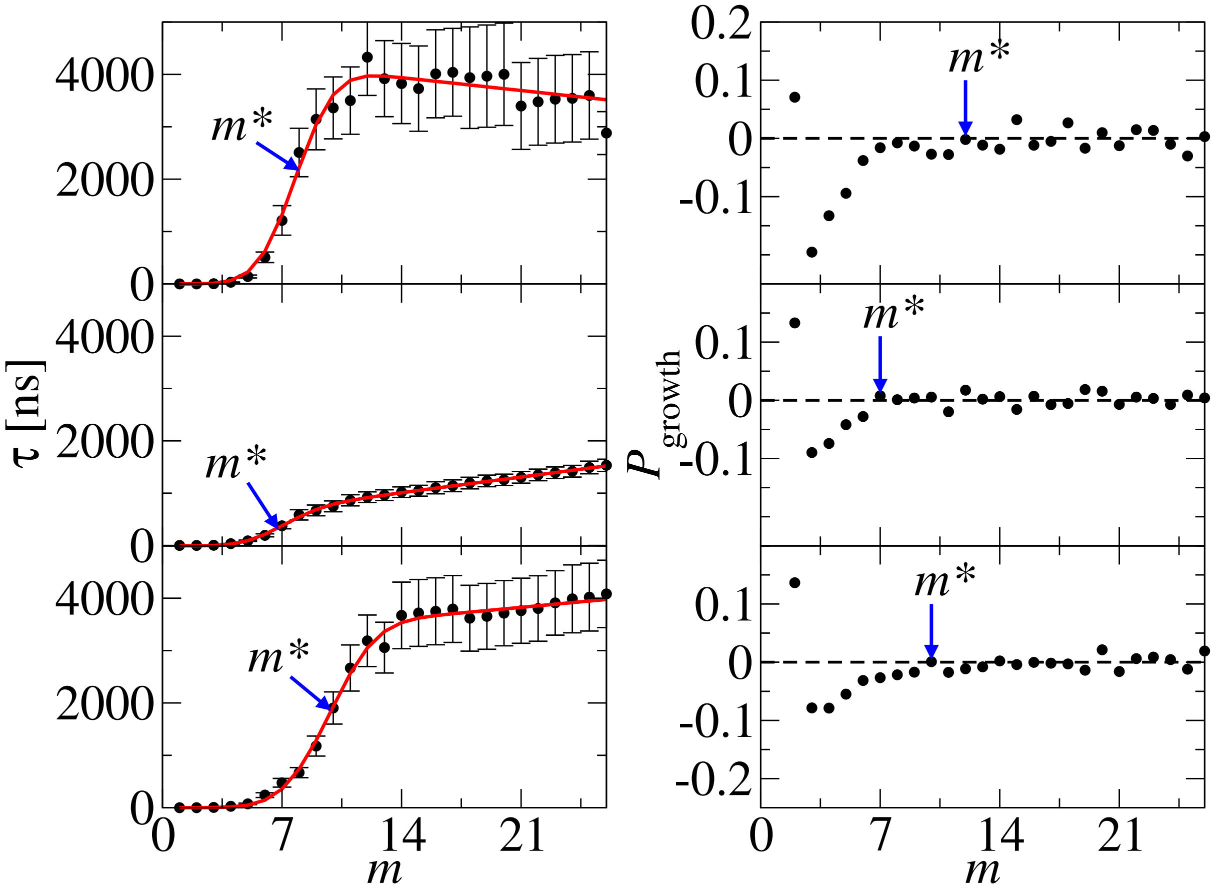


Figure S1: Analysis of the critical nucleus size for three systems: $\varepsilon = 1.4$ kJ/mol, $k_\theta = 400$ kJ/mol (top panels), $\varepsilon = 1.5$ kJ/mol, $k_\theta = 100$ kJ/mol (middle panels), and $\varepsilon = 1.6$ kJ/mol, $k_\theta = 10$ kJ/mol (bottoms panels). The mean first-passage times, $\tau$, as a function of the largest cluster size, $m = M_{\max}$, are shown on the left. The black circles represent the simulation data, whereas the red line is given by Eq. 5 in the main text. The right panels show the growth probability, $P_{\text{growth}}$, as a function of the largest cluster size, $m = M_{\max}$, calculated from transition probability matrix. The black circles represent the simulation data, the dotted line, added for clarity, shows the growth probability equal to 0.

.

## S2 Aggregation kinetics vs. transient aggregate morphologies

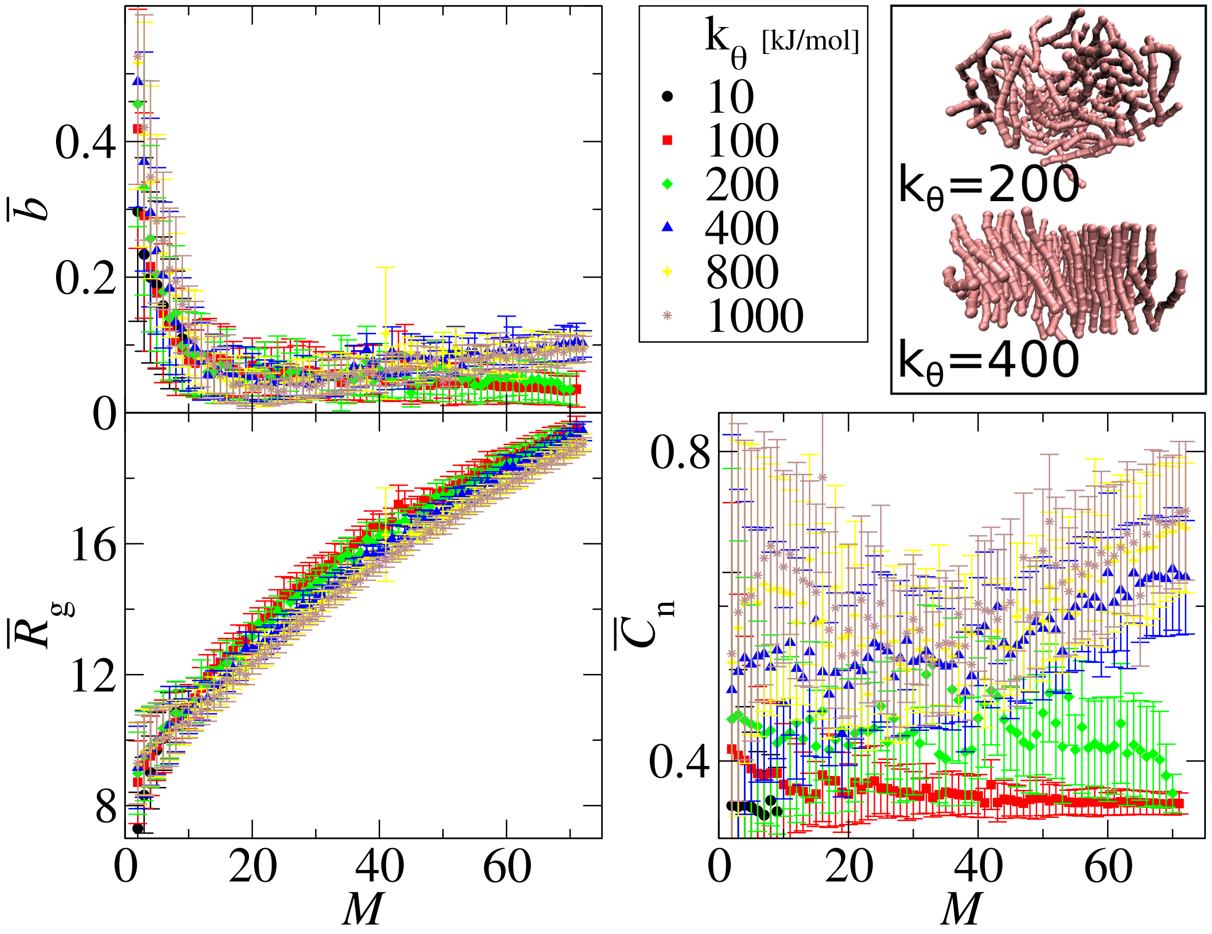


Figure S2: Three structural parameters as functions of the aggregate size, $M$: the average asphericity, $\overline{b}$ (upper left panel), average radius of gyration, $\overline{R}_{\rm g}$ (bottom left panel), and average end-to-end correlation parameter, $\overline{C}_{\rm n}$ (bottom right panel). The data is presented for systems with variable chain stiffness, $10 \le k_\theta \le 1000$ kJ/mol, as indicated. The interaction strength is constant, $\varepsilon = 1.5$ kJ/mol. The right upper panel show the sample structures of final aggregates for two chain stiffnesses, $k_\theta =$ 200 kJ/mol (upper) and $k_\theta = 400$ kJ/mol (bottom).

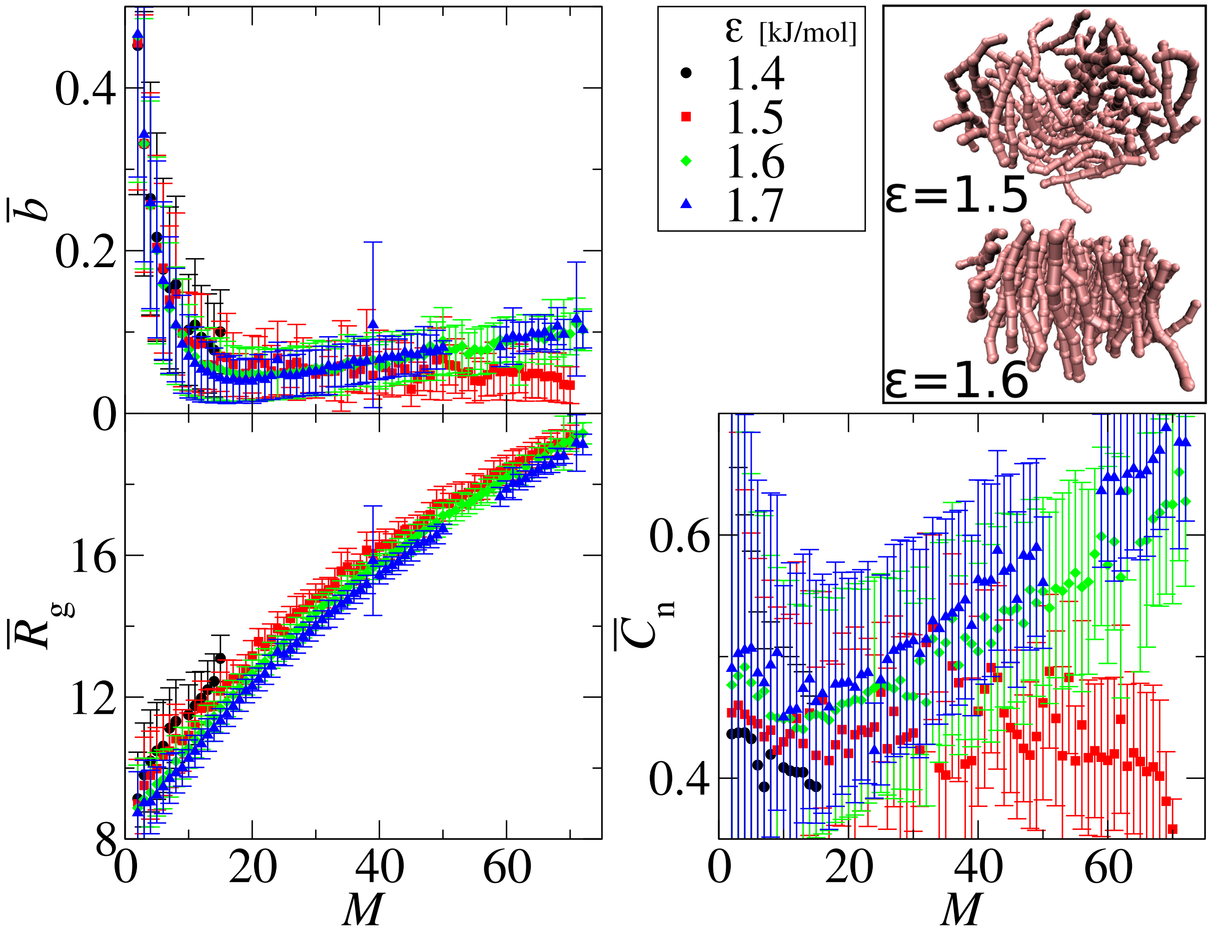


Figure S3: Three structural parameters as functions of the aggregate size, $M$: the average asphericity, $\bar{b}$ (upper left panel), average radius of gyration, $\overline{R}_{\mathrm{g}}$ (bottom left panel), and average end-to-end correlation parameter, $\overline{C}_{\mathrm{n}}$ (bottom right panel). The data is presented for systems with the variable interaction strength, $1.4 \leq \varepsilon \leq 1.7$ kJ/mol as indicated. The chain stiffness is constant, $k_\theta = 200$ kJ/mol. The right upper panel show the sample structure of final aggregates for $\varepsilon = 1.5$ kJ/mol (upper) and $\varepsilon = 1.6$ kJ/mol (bottom).

## S3 Aggregation of long chains, $N_{SA} = 16$

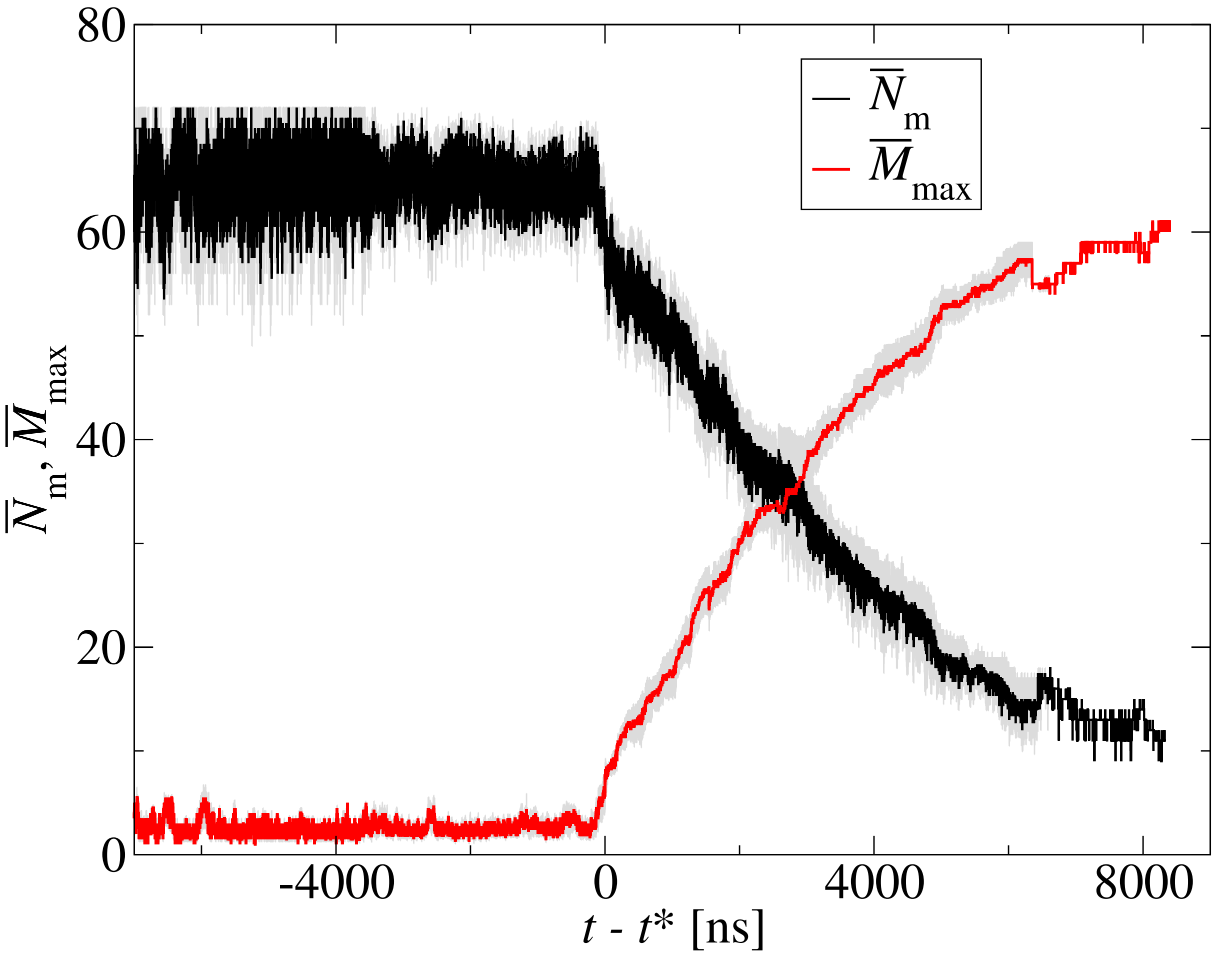


Figure S4: The monomer (black line) and the largest cluster (red line) kinetic curves for system with long peptides, $N_{SA} = 16$. The interaction strength is $\varepsilon = 1.0$ kJ/mol and the chain stiffness is $k_\theta = 1000$ kJ/mol.

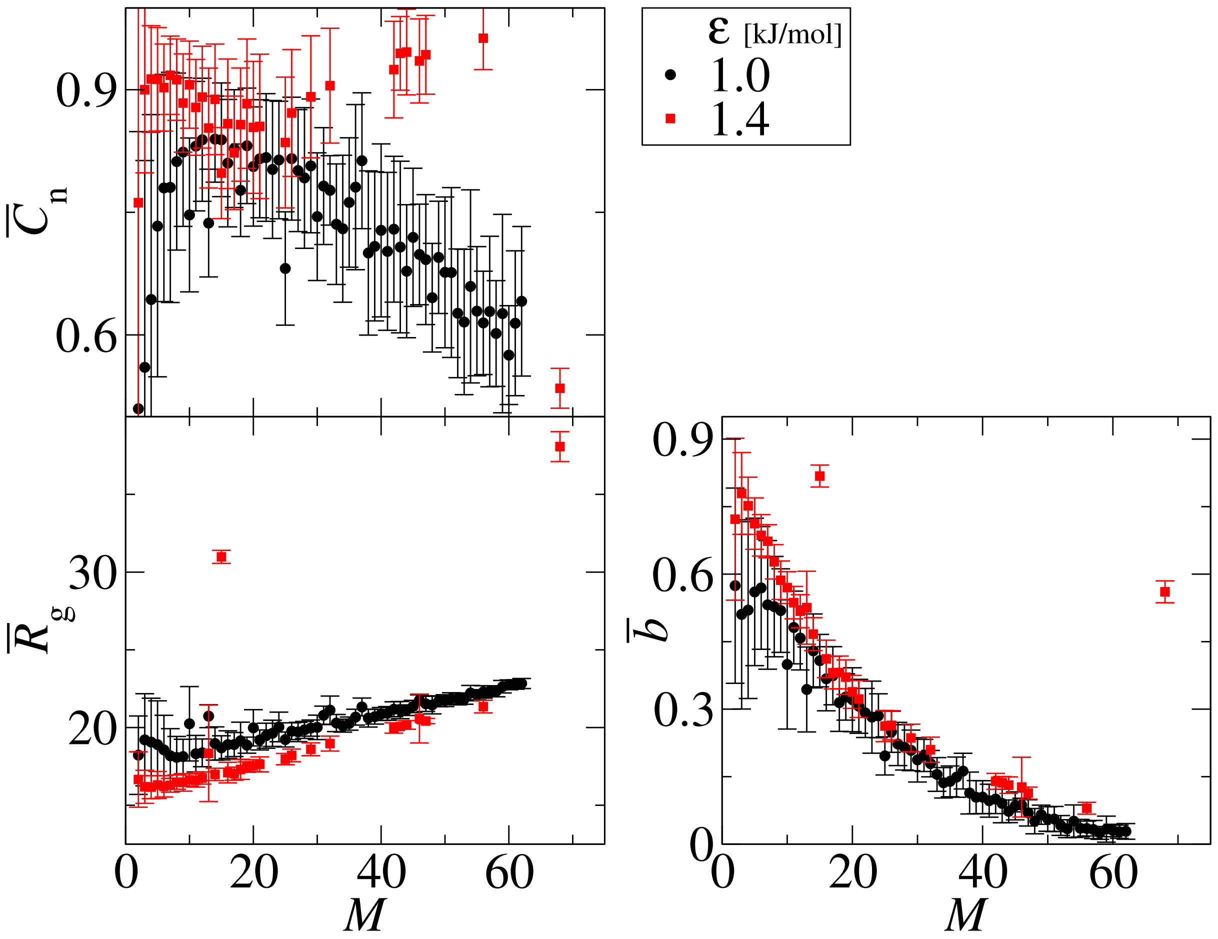


Figure S5: Three structural parameters as a function of the aggregate size, $M$: the average asphericity, $\overline{b}$ (bottom right panel), average radius of gyration, $\overline{R}_{\mathrm{g}}$ (bottom left panel), and average end-to-end correlation parameter, $\overline{C}_{\mathrm{n}}$ (upper left panel), for systems with long peptides, $N_{\mathrm{SA}} = 16$, and variable interaction strength, $\varepsilon = 1.0$ kJ/mol and $\varepsilon = 1.4$ kJ/mol, as indicated. The chain stiffness is constant, $k_\theta = 1000$ kJ/mol.

## S4 Movies illustrating the aggregation modes

Three types of kinetic behaviors are presented in the movies:

- eps1.4_k200.mpg shows no-aggregation for peptides with $\varepsilon = 1.4$ kJ/mol and $k_\theta = 200$ kJ/mol.
- eps1.5_k200.mpg shows the nucleated aggregation for peptides with $\varepsilon = 1.5$ kJ/mol and $k_\theta = 200$ kJ/mol. The lag phase ends around 25 s and then the formation of one, disordered aggregate is seen.
- eps2.0_k1000.mpg shows the downhill aggregation for peptides with $\varepsilon = 2.0$ kJ/mol and $k_\theta = 1000$ kJ/mol. The transient oligomers and mature aggregates are ordered.
